# One Health approach to mental health- mental health recovery in times of crisis: conference proceeding

**DOI:** 10.1186/s12919-026-00389-x

**Published:** 2026-08-12

**Authors:** Surafel Worku Megersa, Yothor Tesfaye Balcha, Munit Abdulreshid Abdullahi, Wolfgang Krahl, Nora Krahl, Tarek Jebrini, Johanna Mahr-Slotawa, Andrea Jobst

**Affiliations:** 1 CIHLMU Center for International Health, LMU University Hospital, LMU Munich, Munich, Germany; 2 Department of Psychiatry, Saint Paul’s Hospital Millennium Medical College, Addis Ababa, Ethiopia; 3 Yekatit 12 Hospital Medical College, Addis Ababa, Ethiopia; 4 International Network for cooperation in Mental Health, Munich, Germany; 5 Department of Psychiatry and Psychotherapy, LMU University Hospital, LMU Munich, Munich, Germany; 6 TH Rosenheim, Department of Health, Rosenheim, Germany

**Keywords:** Mental health, One Health, Innovative approach, Crisis, Recovery, Ethiopia

## Abstract

**Background:**

Globally, mental health disorders are on the rise in response to both natural and man-made crises. Especially in low and middle-income countries (LMICs) like Ethiopia, this is further stretching the already limited mental health facilities, necessitating an innovative solution to the challenges it presents. With the primary goal of examining methods to enhance the quality and accessibility of mental health care in Ethiopia, the Center for International Health at the University hospital of Ludwig Maximilian University Munich (CIH^LMU^), the International Network for Cooperation in Mental Health (i.nez), and Saint Paul’s Hospital Millennium Medical College (SPHMMC) collaborated to organize this 2024 conference, which was held in Addis Ababa, Ethiopia.

**Conference summary:**

The conference focused on three key themes: *(Theme I) rehabilitation and recovery, (Theme II) a One Health approach to conflict response, and (Theme III) innovative strategies for mental health recovery.*

The event featured a number of presentations, panel discussions, and networking opportunities bringing together important stakeholders, including representatives from ministries, professional associations, government and non-government service providers, service users, caregivers of individuals with mental health conditions and journalists.

**Conclusion:**

A central takeaway from the conference was the urgent need for multi-sectoral collaboration to enhance mental health services and ensure more accessible, high-quality care across Ethiopia.

## Introduction

The “One Health Approach to Mental Health—Mental Health Recovery in Times of Crisis” conference, held on December 6 and 7, 2024, in Addis Ababa, Ethiopia, marked a significant milestone in exploring the role of One Health (OH) approach in addressing mental health challenges, particularly in LMICs like Ethiopia. This event was the second in a series of conferences organized through a collaborative effort by the CIH^LMU^ and its affiliated Global Mental Health Research Group within the Department of Psychiatry and Psychotherapy at the University Hospital of Ludwig Maximilian University, Munich, Germany, i.nez, Germany, and the Department of Psychiatry at SPHMMC, Addis Ababa, Ethiopia.

Globally, mental disorders are the leading cause of years lived with disability (YLD), with one in every eight people in the world living with a mental disorder [1] and one in ten global burdens of disease attributed to common mental disorders (CMD) [2]. Mental health disorders are linked to a decreased quality of life, impaired functioning in daily activities such as work, education, social interactions, and self-care, as well as a reduced life expectancy [1]. A community-based cross-sectional study in Ethiopia found the prevalence of CMDs to be around 24.7% [3]. Despite the significant mental health care needs in LMICs, access to mental health services remains limited for various reasons [4]. This highlights the urgent need for timely and tailored mental health support and care. The conference had three key themes: mental health rehabilitation and recovery (Theme I), conflict response through a One Health approach (Theme II), and innovative strategies for mental health recovery (Theme III).

In Ethiopia, ongoing natural and man-made crises, including external and internal conflicts have been linked to an increase in mental health disorders, with people in affected areas reporting higher levels of anxiety, depression, and post-traumatic stress disorder (PTSD), along with overall poor mental well-being. This further stretched the already scarce mental health resources within the country, highlighting the need to find innovative ways to support mental health recovery and for a comprehensive response that incorporates mental health into wider health (One Health) and humanitarian initiatives.

In the context of mental health, the One Health approach goes beyond the management of classic zoonotic disease to address the socio-ecological determinants of psychological well-being in a holistic manner [5]. In agrarian and pastoralist societies such as those in Ethiopia, natural and man-made crises not only damage infrastructure, but also devastate ecosystems, displace human populations and disrupt livelihoods dependent on livestock. The distraction of environmental and economic fabric can trigger severe psychological trauma, depression and anxiety [6].

Adopting a One Health approach to mental health recovery recognizes that sustainable psychological healing cannot occur in isolation, but must be coupled with environmental restoration, animal welfare, and cross-sectoral community support systems.

The One Health approach is globally relevant, as it recognizes the interconnectedness of human, animal, and environmental health and promotes coordinated, cross-sectoral responses to complex health challenges worldwide.

Key stakeholders across different regions of the country, such as ministry representatives, hospital administrators, professional associations, researchers, practitioners, service providing governmental and non-governmental institutions, people with lived experience, caregivers of people with mental illness, and journalists from national media were invited to attend the conference with the main aim of providing a platform to exchange knowledge, share experience and develop strategies to improve the overall quality of mental health services in Ethiopia.

## Methods

### Institutional framework

This is the second Mental Health Conference organized under the umbrella of the One Health Approach. In 2023, the first conference was held in Addis Ababa and was funded by the German Developmental Exchange Service (DAAD) and jointly organized by CIH^LMU^ and the Department of Psychiatry at SPHMMC. The conference focused on a One Health approach to child and adolescent mental health. In addition to multiple mental health professionals from Germany and Ethiopia, other key stakeholders – including politicians, human rights activists, journalists, and young people with lived experiences – participated in the conference.

A close partnership, grounded in personal commitment and long-standing professional relationships, has been established since 2014 between the Department of Psychiatry and Psychotherapy of LMU University Hospital Munich, its Global Mental Health Research Group, i.nez, and the Department of Psychiatry at SPHMMC. Lecture exchanges, academic opportunities, jointly conducted projects, regular virtual case discussions, and mentorship programs are in place. Regular Zoom conferences, held since 2023 and occurring up to twice monthly, have ensured strong communication and progress of the joint activities. This conference aimed to further deepen the existing collaboration and create opportunities for networking, knowledge exchange, and overall improvement in the quality of mental health services.

### Organizational framework

The organizing committee for the 2024 One Health conference comprised three consultant psychiatrists – two from Germany and one from Ethiopia and two general practitioners from Ethiopia. The members of the organizing committee have a long-standing collaboration at both institutional and personal levels.

### Content selection

The organizing committee identified three key, practical themes: rehabilitation and recovery (Theme I), the One Health approach to conflict response (Theme II), and innovative strategies for mental health recovery (Theme III), in light of ongoing natural and man-made crises and their impact on the prevalence and delivery of mental health services in Ethiopia. A carefully selected group of potential speakers and panelists was assembled based on professional expertise and relevance to the conference topics. This group included professionals from conflict-affected regions as well as other key stakeholders who play a critical role in mental health service provision.

Potential speakers and panelists were formally invited and provided with the event's thematic objectives, along with specific topic for their presentations, following initial contact and confirmation of their participation.

### Conference delivery

Online registration for the conference was free and remained open from October 15 to November 10, 2024. The conference was held in person from December 6th to 7th, 2024, at the Grand Palace Hotel in Addis Ababa, Ethiopia, while online speakers participated via the Zoom platform.

A total of 70 individuals registered for the conference, with 64 ultimately attending. Of these, 11 participants came from various Ethiopian regions, including Mekelle, Jimma, Gondar, and Hawassa, while five attendees joined from Germany. The remaining participants were based in Addis Ababa. The event was moderated by consultant psychiatrists from SPHMMC and CIH^LMU^ on behalf of the organizing team. The conference agenda included several segments: opening remarks, keynote presentations, a panel discussion, and closing remarks.

The conference was opened with a welcome from Andrea Jobst-Heel, lead of the organizing team. Following this, remarks were delivered by Selamawit Ayele, Head of the Non-Communicable Diseases and Mental Health Desk at the Ministry of Health of Ethiopia. Sandra Dehning, a child and adolescent psychiatrist from Germany, also extended her greetings virtually.

On the first day of the conference, under the theme of ‘Rehabilitation and Recovery’, four experts gave presentations – three in person and one online. Each speaker had approximately 30 min, including time for questions and answers.

Following the presentations, a panel discussion was held, moderated by a consultant psychiatrist from SPHMMC and member of the conference organizing committee (SW), incorporating queries and contributions from participants. Five panelists participated in the discussion, which lasted 45 min.

In the afternoon session of day one, under the theme “One Health Approach to Conflict Response”, four experts gave presentations –three in person and one online. Each speaker had approximately 30 min, including time for questions and answers.

Following the speakers' presentations, a panel discussion was held, moderated by a consultant psychiatrist from CIH^LMU^ and lead of the conference organizing committee (AJ), incorporating queries and contributions from participants. Five panelists took part in a discussion, which lasted 45 min.

On day two of the conference, under the theme “Innovative Approaches to Mental Health Recovery”, six experts gave presentations –five in person and one virtual. Each presenter had 30 min, including time for questions and answers.

The conference concluded with closing remarks by Wolfgang Krahl, and certificates of attendance were issued to all participants and speakers.

## Summary of presentations

### Day one

#### Theme: rehabilitation and recovery

##### Integrating one health to rehabilitation programs—the key to sustainable recovery

Wolfgang Krahl, MD, MA, consultant Psychiatrist, chairman of i.nez, and external Consultant at CIH^LMU^

**Overview**

One Health is not a new concept; it has been taught already in 1978 at the University of Heidelberg in Germany. However, it has gained renewed attention and evolved significantly over the past decade due to the increased frequency and severity of threats that link the health of humans, animals, plants, and the environment. The One Health approach emphasizes a holistic, systems-based perspective that recognizes the interconnection of human, animal, plant and environmental health [7, 8]. Health, including mental health, can also be understood through a holistic bio-psycho-social-cultural lens. Physical and mental well-being are shaped by social determinants as poverty, unemployment, pollution, climate change, and war, with zoonotic diseases such as Coronavirus disease [9] further illustrate the close relationship between animal health and human well-being.

Rehabilitation plays a critical role for individuals living with chronic and disabling conditions, including schizophrenia and substance use disorders [10]. Although psychosocial rehabilitation services have expanded globally, many LMICs continue to face major gaps in health and social service provision. As a result, the responsibility of care often falls heavily on family caregivers [11], a burden that is further exacerbated by urbanization and the erosion of traditional family support systems.

Regarding care settings, community-based care is widely regarded as superior to institutional care in promoting treatment acceptance, social inclusion, and recovery [12]. Successful examples include Nigeria’s Aro village system, which integrates traditional healers; Malaysia’s Farm Project, which integrates people living with schizophrenia through meaningful employment; Germany’s social firms that provide inclusive work opportunities; India’s initiatives addressing homelessness and mental-health -related stigma and Indonesia’s efforts to reduce the use of physical restraints through improved training and strengthened community mental health services.

In conclusion, safeguarding the human rights of individuals with mental health conditions requires the promotion of locally developed rehabilitation programs, enhanced community integration, and sustained investments in healthcare systems.

**Discussion**

Policies frequently overlook broader determinants that negatively affect health, including adequate public transportation and poor consideration of animal welfare in urban development. Despite their substantial contribution to the global burden of disease, substance use disorders remain underprioritized in global mental health agendas. This neglect is particularly evident among young people, both in Africa and worldwide, where the prevalence and impact of substance use disorders continue to grow. Furthermore, caregiving responsibilities disproportionately falls on families, especially in rural settings, with limited resources and service availability, exacerbating health inequities and reinforcing existing social and economic disparities.

##### Innovative rehabilitation programs for addiction

Etsedingl Hadera, BSc, MSc, a mental health specialist at Mekelle University, Ayder Specialized Hospital

**Overview**

In 2016, an out-patient addiction rehabilitation program for individuals living with substance use disorders was established in Mekelle, Tigray, Northern Ethiopia. The program provides comprehensive assessments and tailored support, including both individual and group therapy, as well as occupational and spiritual interventions, reflecting a holistic approach to care. The program was severely disrupted during the Tigray conflict (2020–2022), which caused widespread death, displacement, and further traumatic experiences. Substance use increased, including new patterns such as tramadol and cannabis use, even among school children. Additionally, services were limited, and the infrastructure needed rebuilding. Post-conflict, efforts to rebuild the rehabilitation program focused on reconnecting with collaborators and capacity-building through training professionals in various parts of Tigray in partnership with Mekelle University.

Local solutions – supported by advocacy and school-based awareness campaigns – were emphasized as key strategies to address rising substance use in Tigray. In conclusion, the program’s resilience, together with the dedication of its team, has been vital in sustaining this essential service during a period of hardship, with a clear commitment to building back better.

**Discussion**

The challenges faced during and after the conflict – including the disruption of the rehabilitation program and the overall health care system, as well as the ongoing rebuilding efforts – were highlighted. The alarming increase and changing patterns of substance use in Tigray following the conflict were also discussed. The participants acknowledged the resilience of the professionals serving under these difficult circumstances and emphasized the need for a collaborative approach.

##### Integrating gardening as a therapeutic tool for a rehabilitative program

Workenesh Tessema, BSC, MSc, Assistant Professor, an occupational therapist at Jimma University

**Overview**

Occupational Therapy (OT) is a skill-based intervention that integrates art and science to help individuals regain and enhance their ability to perform daily activities. According to the World Federation of Occupational Therapists, 2010, OT aims to promote independence among individuals who are at risk of, or experiencing, challenges arising from injury, disease, impairment, disability, or activity limitations. OT supports rehabilitation and recovery for individuals across various age groups and conditions, including mental health conditions such as depression, schizophrenia, and substance use disorders, with personalized approaches tailored to each person’s unique needs.

The goals of OT are holistic, emphasizing the preventing of prolonged hospitalization, support for recovery, mobilizing of individual strengths, building confidence, and the promotion of healthy work and leisure habits. It can be provided in various care settings, including hospitals, rehabilitation centers, substance use recovery centers, and special educational needs schools, addressing skills like independent living, socialization, employment, and parenting.

The OT process involves several key steps: referral, screening, evaluation, treatment planning, implementation, re-evaluation, discharge, and conclusion of care. Important factors in OT include the client and their family, the therapist, the environment, and the tasks involved. OT activities may include woodworking, activities of daily living, crafts, arts, music, poetry, weaving, and social skills training. A crucial aspect of OT is actively involving individuals in selecting activities that build on their strengths and offer opportunities for new experiences. The therapist’s role is flexible and may include acting as a supporter, guide, teacher, friend, or parental figure, depending on the client´s needs.

In conclusion, OT is an effective approach to rehabilitation and recovery, particularly for those living with mental health conditions, and advocating for its inclusion in mental healthcare and recovery is essential.

**Discussion**

Emphasizing client-centered OT empowering individuals to choose activities and take ownership of their recovery was identified as a key factor in the therapy’s success. Interestingly, the landscape of OT in Ethiopia is expanding, as evidenced by a recent establishment of an OT program at the university of Gondar. This highlights the need to advocate for non-pharmacological approaches to mental healthcare and recovery, such as OT, and to incorporate activities that consider the local context, while providing employment opportunities.

##### Integrating holistic Well-Being Therapy (WBT) in day clinic treatment

Tarek Jebrini, MD, Psychologist, Psychiatry resident at LMU Clinic, Department of Psychiatry and Psychotherapy

**Overview**

Well-Being-Therapy (WBT) focuses on enhancing well-being rather than solely reducing symptoms, through an integrative, transdiagnostic clinical approach. In doing so, WBT closely aligns with the World Health Organization´s recommendation that well-being – alongside symptom remission – is essential for achieving mental health and recovery. Goals of WBT include promoting resilience, activating personal resources, fostering psychological flexibility, and preventing relapse. WBT is not intended as a standalone intervention but is designed to be used in combination with other therapeutic approaches. It is a short-term therapy model, typically consisting of eight or fewer sessions, and incorporates established, evidence-based strategies from Cognitive Behavioral Therapy (CBT) alongside value-based and growth-oriented perspectives.

The therapy strives to promote balance across six key dimensions of well-being that are relevant to the patient´s life, including autonomy, personal growth, purpose in life, self-acceptance, positive relationships and environmental mastery. WBT has shown promising outcomes for different forms of anxiety and affective disorders in both adults and children [13–16].

The implementation of WBT in a psychiatric day clinic – currently practiced at the Department of Psychiatry and Psychotherapy of the University of Munich – consists of a multimodal program by a multidisciplinary team. This team includes physicians, psychologists, nurses, occupational therapists, sociotherapists, physiotherapists, and music therapists, all of whom are trained in the application of WBT. The WBT program includes both group and individual sessions. An accompanying study evaluating the introduction of WBT in the day-clinic setting is currently underway, with the primary aim of assessing its feasibility, practicability, and acceptance.

**Discussion**

Emphasizing the importance of adopting innovative therapeutic approaches, the opportunities and potential challenges of implementing WBT in an Ethiopian context were discussed. It was highlighted that the dimensions of WBT may require cultural contextualization and reframing.

**Panel discussion I**

***Participatory approach with-in mental health services***

Moderator: Surafel Worku

Panelists: Wolfgang Krahl; Johanna Mahr-Slotawa (Ph.D. in public health and specialized on young people participation, TH Rosenheim); Meskerem Abebe (MD, Assistant Professor of Psychiatry, Head of Department of Psychiatry at SPHMMC); Yodit Tesfaye- (A teacher and a representative of the Mental Health Service Users’ Association (MHSUA) in Ethiopia, People with Lived Experience (PWLE,); Kassahun Tadesse (A caregiver for a child with developmental disorder, successfully completed the World Health Organization’s Caregiver Skills Training (WHO’s -CST)

The participatory approach in mental health services is defined by the active involvement of service users in all aspects of their care, shifting away from traditional, top-down models. This approach is based on the core principle of empowering service users by understanding their needs and enabling them to voice their perspectives. It recognizes that individuals with lived experience are experts in their own needs and should play a central role in shaping services. A key element of the participatory approach is the fostering of a collaborative partnership between professionals and service users, moving away from hierarchical relationships.

This approach requires a shift in the dynamics of mental health services. The traditional model, where professionals hold authority, is replaced by a collaborative model where clients are active partners. In a participatory model, professionals become facilitators and supporters, actively listening to clients’ needs, preferences, and goals and incorporating them into treatment plans. This allows clients to make informed decisions and take ownership of their recovery. Furthermore, clients benefit from treatment and learn from peers, potentially taking on therapeutic roles for each other.

Several key elements are necessary to implement a participatory approach. Firstly, both professionals and organizations need to shift from a paternalistic model of decision making to a participatory one. This transformation should be supported by providing training and resources to professionals, enabling them to facilitate participatory processes effectively. Secondly, creating opportunities for service users to share their experiences is crucial, which can be achieved through focus groups, advisory boards, and peer support programs. Lastly, the approach must be culturally sensitive and inclusive, respecting the diverse backgrounds, cultures, and religious contexts of the service users.

Implementing a participatory approach involves various stakeholders. MHSUA representatives share their experiences, participate in decision-making, and offer peer support. Professionals are tasked with listening, facilitating, and empowering service users in their treatment, moving away from a purely directive role, and must be culturally sensitive. Families play a crucial role in supporting patient participation, and their perspectives need to be respected. Institutions and policymakers are responsible for providing resources, and workshops, and creating policies that support a participatory approach.

The participatory approach offers numerous benefits, including increased engagement in services as service users feel more comfortable and understood. It empowers service users by encouraging active involvement in treatment, which can lead to improved outcomes. It also ensures culturally relevant services that are more appropriate and effective, facilitating personalized care that is tailored to individual needs and preferences, while promoting better relationships between service users and professionals.

Engaging families, communities, and policymakers is crucial. Training and workshops for professionals are necessary, and it's important to mobilize resources and incorporate cultural considerations in mental health service delivery. Regular monitoring and evaluation should be conducted to ensure effectiveness. Developing and expanding peer support programs and actively engaging with communities are essential, along with raising awareness and acceptance through various dissemination methods.

Specifically, regarding children’s participation in mental health services, participation is defined based on Article 12 [1] of the UN Convention on the Rights of the Child. It states that every child capable of forming their own views has the right to express those views freely in all matters affecting them, and that their views must be given due weight. In mental health services, this principle can be realized through participatory approaches that involve young people in shared decision-making, empower them, and foster mutual learning.

The panelists identified three main ways in which children’s participation can be implemented in mental health services:

1. Service Delivery – Children act as experts with lived experience. For example, in the United Kingdom (UK), peer-to-peer treatment models have been established, and youth committees are involved in service planning. In Michigan, United States of America (USA), peer awareness programs empower young people to support one another through school-based mental health campaigns.
2. Informing Institutions and Services – Children share their views to help shape and improve services. For instance, when planning a new youth mental health ward at SPHMMC, involving children from the beginning could help ensure their needs are understood. In the UK, for example, children’s perspectives contribute to institutional transformation.
3. Participatory Research – Young people and adults collaborate on research projects. The findings are used to improve mental health services, and in some cases, children develop their own recommendations for service delivery, such as in Kenya.

It is important to emphasize that children’s participation in mental health services is still a developing field, with significant potential for further practical exploration and innovation.

##### Response recovery resilience project

Andrea Jobst-Heel- MD, Consultant Psychiatrist, Postdoctoral at LMU Clinic, Department of Psychiatry and Psychotherapy

**Overview**

Traumatic experiences are widespread, particularly in conflict-affected communities, where the prevalence of Post-Traumatic Stress Disorder (PTSD) can reach up to 60%, profoundly affecting key domains of everyday life. Trauma and aggression are closely intertwined in conflict and post-conflict settings. Exposure to trauma can fuel violence through both reactive and appetitive aggression, creating a vicious cycle that perpetuates collective violence, including domestic and community-based violence [17]. Moreover, trauma is transgenerational: it can be transmitted from one generation to the next, shaping the values, behaviors, and decision-making of entire communities and their future leaders [18]. For these reasons, trauma-informed interventions are essential and should be systematically integrated into peacebuilding efforts. Evidence consistently shows that trauma-focused interventions are more effective than non-trauma-focused approaches in treating trauma-related disorders [19, 20]. Such interventions should be made available to both civilians and combatants and should include the assessment and treatment of perpetrators of violence. One well-established trauma-focused intervention is Narrative Exposure Therapy (NET), developed by Maggie Schauer, Frank Neuner and Thomas Elbert at the University of Konstanz, Germany. NET is a short-term intervention, typically delivered over 5 to 12 sessions, that supports individuals in processing traumatic experiences by constructing a chronological life narrative. This process links fragmented and overwhelming traumatic memories with neutral, non-traumatic experiences, thereby restoring temporal and spatial context and promoting integration of the traumatic events. Implemented in over 30 countries across diverse post-conflict settings, NET has demonstrated strong evidence in reducing trauma-related mental health symptoms, appetitive aggression, and ongoing violence, while also contributing to improved social cohesion [21].

**Discussion**

Experiences from Ethiopia illustrate the profound and pervasive impact of trauma in conflict-affected populations. Preliminary findings from a needs assessment conducted in five regions in November 2023 indicate that survivors of Gender-Based Violence (GBV) had been exposed to multiple traumatic events and diverse forms of GBV, alongside high levels of PTSD, major depressive disorder, and appetitive aggression. Service providers reported that many clients had histories marked by childhood adversities, repeated exposure to GBV, cumulative traumas, and war-related experiences. These findings underscore the urgent need for targeted, trauma-informed interventions aimed not only at the treatment but also at preventing the intergenerational transmission of trauma. The critically high burden of trauma in conflict-affected settings highlights the necessity of integrating trauma-informed approaches as a core component of humanitarian, protection, and peacebuilding responses.

##### One health alliance for conflict response

Rahwa Berhe- Founder and country director of One Health and Economic Resilience Initiative

**Overview**

The presenter discussed the trauma experienced by individuals, emphasizing the importance of testimonies and firsthand accounts from survivors. Key questions revolved around feelings of exploitation related to their trauma. The fragmentation in addressing trauma was identified as a major issue, with affected individuals facing challenges in financial, health, and environmental areas, such as livestock looting and zoonotic infections. Livestock is crucial for agriculture through soil enrichment.

A proposed solution is an integrated recovery program in Ethiopia, initiated by the One Health Alliance founded in 2024, involving partners like LMU and Mekelle University. Discussing the importance of economic empowerment and environmental health for post-conflict recovery, Berhe emphasized the integrated approach of the Alliance, which addresses the interconnectedness of human, animal, and environmental health. The approach involves identifying participants, implementing integrated interventions, allowing participant choices, and ongoing monitoring.

The program targets conflict-affected regions like Tigray, Amhara, and Afar, with expansion plans. It features an innovative framework for post-conflict recovery and self-regeneration funding. Progress includes applications to various funding bodies, with aspirations to extend outreach globally. The presenter encouraged widespread involvement in this initiative.

**Discussion**

Explanation was given on the objectives and targets of the project, highlighting it as a response to the lack of sustainable efforts in addressing trauma. Questions were raised about why the focus is specifically on women affected by war rather than other women. The speaker acknowledged the concern and clarified that the initial assessment targeted these women, but the project plans to eventually expand to include others as well.

##### Economic empowerment and environmental health as key aspects for post-conflict recovery: the case of Desa’s Forest Management

Yemane Gebru- WeForst, regional manager, Tigray

**Overview**

Environmental health is crucial in post-conflict recovery, as natural resources are often devastated by deforestation, soil degradation, and weakened ecosystems, increasing vulnerability. Addressing environmental health is key to recovery. WeForest is an international Non-Profit Organization (NGO) focusing on global forest restoration and protection. In Ethiopia, its project in Desa’a Forest (located between the Tigray and Afar regions) aims to restore an area that lost 40% of its forest between 1972 and 2018, while addressing the high poverty rate among local communities. The Tigray conflict (2020–2022) further disrupted the region’s economic and social stability. The Desa’a Forest project combines reforestation with livelihood rebuilding, offering job opportunities in planting and seedling care, as well as training and material support to promote self-reliance and entrepreneurship. Over 10,000 households are involved, with a focus on empowering women and landless youth through small businesses in beekeeping, raising chickens and sheep, and growing high-value tree seedlings, to support economic recovery and reduce aid dependency. The project also focuses on rehabilitating degraded land, addressing water scarcity, and preventing social erosion with stone and soil terraces, and promoting renewable energy through efficient cookstoves and solar lighting. At a higher level, the project works on strengthening governance and policy at the local level, encouraging community mobilization, and engaging government and NGOs both locally and internationally. The goal is to restore over 3,800 hectares and engage over 10,900 households by 2030.

**Discussion**

The link between environmental health and economic empowerment plays a crucial role in rehabilitating post-conflict regions like Tigray. By creating jobs, providing skills training, and promoting sustainable livelihoods through reforestation efforts, the project helps communities both their environment and economy. These initiatives not only support ecosystem restoration but also generate income opportunities, contributing to long-term recovery and resilience.

##### One health need of conflict-affected populations

Haish Desta- MD, Assistant Professor of Psychiatry, head of the department of Psychiatry, Mekelle University, Ayder specialized hospital

**Overview**

Hundreds of thousands of people have died because of the internal conflict in Ethiopia's Tigray region in recent years, leading to severe political, economic, and social repercussions. This has had a significant negative impact on the healthcare system. Stressors such as sexual abuse, job loss, interrupted education, perceived threats, lack of social support, displacement, food insecurity, and medication shortages have all contributed to the rapid rise in the prevalence of mental disorders.

Common mental health issues include high suicide rates, addiction, post-traumatic stress disorder (PTSD), depression, and anxiety. Professionals in the area have collaborated with NGOs, to offer mental health and psychosocial support training, engaging the community, religious leaders, and the media to maintain routine clinical services despite severe shortages. Additional challenges include a non-conductive work environment due to ongoing conflict, a shortage of medications, and a lack of professionals, as many have resigned. There were no clinical psychologists available, and forensic psychiatry services were not provided at all. Mekelle University is currently working on the establishment of an addiction treatment facility and a residency program in psychiatry.

**Discussion**

The participants recognized the challenges faced during conflict and after the conflict, acknowledged the resilience of the professionals working under these difficult circumstances, and emphasized the importance of collaborative efforts in the rebuilding process. A critical One Health need in this context is the integration of mental health with primary care, ensuring that both physical and psychological needs of conflict-affected populations are addressed simultaneously. Strengthening community-based mental health services, along with coordination between healthcare, social support, and environmental resources, is essential to promote holistic recovery and resilience among survivors.

**Panel discussion II**

***One health aspects within psychological first aid***

Moderator: Andrea Jobst-Heel

Panelists: Benyam Worku (MD, General and trauma psychiatrist, Associate professor at Addis Ababa University (AAU), cofounder and general manager of Serenity Mental Health), Fasika Amdeselasie (MD, Associate Professor of Surgery, Gastrointestinal and laparoscopic surgeon, Medical educator, Academic quality assurance head at Ayder specialized comprehensive hospital) Hermon Amare (MD, Assistant professor of psychiatry, Senior Advisor for Mental Health and Psychosocial Services at African Medical and Research Foundation (AMREF), Medhin Selamu (Ph.D. Mental health professional and health service researcher at the World Health Organization (WHO)), Fanuel Gashaw (MD, Assistant professor of psychiatry at University of Gondar) and Haish Desta.

The panel discussion adopted a holistic approach to mental health and community well-being, recognizing the interconnectedness of various factors in conflict recovery. It moved beyond traditional, individual-focused mental health interventions to include the social, cultural, economic, and environmental contexts of trauma. This integrated approach seeks not just to treat individual symptoms but to foster long-term community resilience.

A significant portion of the discussion centered on the importance of community-based approaches and cultural appropriateness. The panelists emphasized leveraging existing social structures and traditions such as resuscitating traditional elders or tribal leaders during crisis when formal structures fail. Moreover, the panel explored how cultural practices, beliefs, and traditions could be integrated to foster healing. For instance, the strategic engagement of religious leaders such as local priests assisting children with school reintegration post-conflict illustrates how trusted local actors can facilitate community recovery. The panel recognized that trauma is understood and experienced differently across cultures, and interventions should be tailored to these contexts.

The role of traditional healers emerged as a critical component of the recovery process. In many Ethiopian communities, religious and traditional leaders wield significant influence and trust. Panelists also noted that integrating these leaders into mental health initiatives could be beneficial because they already have the trust of the community. However, they acknowledged potential challenges that could arise with this kind of integration and stressed the need for cultural sensitivity and respect for their roles.

The discussion also delved into the practical aspects of therapy and training, addressing the question of whether to prioritize training lay people or scaling up training for professionals. Although task-shifting can improve access to services, care must be taken to avoid overburdening the remaining healthcare workforce. To facilitate cultural adaptation, integrating native narrative customs, such as peer-to-peer storytelling during traditional coffee ceremonies, offers a non-intrusive way to deliver trauma care. Furthermore, implementing a stepped-care framework ensures that care is closely tailored to the severity of the individual's trauma, while also proactively preventing the duplication of organizational efforts through streamlined institutional coordination. Panelists described a challenge of implementing mental health programs, noting that there were “struggles with cultural adaptation,” and went on to describe how they “use storytelling” and integrate “peer-to-peer story telling over coffee ceremony” as ways to adapt to the culture. The NET approach was considered for its efficacy in conflict settings and it can be used effectively in settings with people who may be uneducated or lay people. Moreover, the use of storytelling and other narrative approaches was recommended as a way to facilitate community healing.

Panelists also discussed strategies for integrating trauma care into existing healthcare systems, with an emphasis on making services accessible in rural and under-resourced areas. The concept of a stepped-care approach was raised, in which individuals receive care tailored to their needs and the severity of their condition, with referrals to specialized care as needed. Additionally, the importance of "avoiding the duplication of efforts and supporting coordination systems,” was discussed indicating that better integration of care could be a way to strengthen service delivery.

Overall, the panel discussion highlighted a move towards a more holistic, integrated, and community-driven approach to mental health in post-conflict settings. By recognizing the interconnectedness of social, cultural, economic, and environmental factors, the panelists advocated for a more comprehensive and sustainable approach to trauma recovery. Panelists also discussed strategies for integrating trauma care into existing healthcare systems, with an emphasis on making services accessible in rural and under-resourced areas. In the discussion it was also highlighted that traditional healers as well as religion are important for many individuals to overcome trauma.

### Day two

#### Theme: innovative approaches to mental health recovery

##### Transcranial Magnetic Stimulation (TMS), Hospital of the Ludwig-Maximilians-University (LMU) Munich| Center for Non-invasive Brain Stimulation Munich (CNBS), An emerging treatment for therapy-resistant depression

Dr. Nora Krahl—MD, Neurologist, Psychiatry resident at LMU Clinic, Department of Psychiatry and Psychotherapy

**Overview**

Transcranial Magnetic Stimulation (TMS) is a non-invasive brain stimulation technique emerging as a treatment for medication-resistant depression. It offers an alternative to medication or invasive treatments with minimal side effects and a non-systemic application. It has shown efficacy, especially for treatment-resistant depression, making it a significant innovation in clinical medicine and neuroscience.

Biophysically, TMS generates a magnetic field through a coil, inducing small electrical currents in targeted brain regions most commonly the left dorsolateral prefrontal cortex (DLPFC), which is often underactive in depression. Repeated stimulation modulates and strengthens neural circuits involved in mood regulation. TMS also promotes neuroplasticity, enhancing the brain's ability to reorganize itself and improve communication between brain regions, such as the prefrontal cortex, amygdala, and hippocampus, ultimately helping to restore balance in brain networks.

TMS is most beneficial for individuals with moderate to severe major depressive disorder without psychotic features, with the best effects seen in patients with treatment-resistant depression, meaning no adequate response to two or more antidepressants or psychotherapy. Exclusion criteria include metal implants in or close to the head, epilepsy or high risk of seizures, and severe or unstable medical and neurological conditions. While there is no evidence of the safety of TMS during pregnancy, it is generally avoided. Beyond depression, TMS has also shown evidence for treating obsessive–compulsive disorder and persistent acoustic hallucinations in psychotic disorders, and potential benefits in PTSD, anxiety disorders, and craving reduction in substance use disorders. The advantages of TMS include that it is non-invasive, does not require anesthesia, has minimal side effects, is an outpatient procedure, and has proven efficacy. It is recommended in the German National Guideline for Unipolar Depression and other international guidelines, such as the American Psychiatric Association (APA) Practice Guideline for the Treatment of Patients with Major Depressive Disorders.

Side effects are typically mild, such as headache or scalp discomfort during or after treatment, brief muscle twitching at the site of stimulation, and very rarely, seizures. Disadvantages include limited accessibility, time commitment, limited efficacy in some patients (40%–50% non-responders), and high treatment costs associated with the device and staff.

When considering the application of TMS in Ethiopia, it is important to consider logistical factors such as maintenance and repairs, the lack of long-term experience with TMS in that context, and patient compliance. There are also cultural barriers, such as stigma, awareness, cultural perceptions, and economic limitations to consider.

**Discussion**

TMS is a promising treatment modality that has the potential to expand the therapeutic landscape in low-resource settings such as Ethiopia. Due to its independence from the continuous supply of psychopharmacological medications and the robustness of the device, which requires minimal technical maintenance, TMS appears to be a sustainable treatment option and a source of hope for individuals with treatment-resistant depression in Ethiopia.

##### The Brain stimulation for Ethiopia’s Advanced Mental Health Care (BEAM) project

Surafel Worku- MD, Assist. Prof. of psychiatry at SPHMMC and a Ph.D. candidate in Medical Research-International Health at CIH LMU

**Overview**

The BEAM project **–** Brain Stimulation for Ethiopia’s Advanced Mental Health Care** –** aims to introduce repetitive Transcranial Magnetic Stimulation (rTMS) as an innovative treatment modality for severe depression in Ethiopia. The project is motivated by the high prevalence of mental disorders, including depression, in Ethiopia and the limited access to mental health services [4]. Additionally, there is an inconsistent availability of essential drugs, and electroconvulsive therapy (ECT) is scarce, costly, and requires specialized training. The collaborative project between the Department of Psychiatry at SPHMMC and the Department of Psychiatry and Psychotherapy at the University of Munich (LMU) seeks to establish the first nationwide – and the first in East Africa – non-invasive brain stimulation unit at SPHMMC procuring the necessary equipment, building staff capacity through training, and eventually scaling up this service across the tertiary healthcare sector nationwide.

Standard treatment involves daily sessions over 3–6 weeks, with session times recently reduced due to technological advances, allowing a single machine to treat 3–4 patients per hour. rTMS is a cost-effective alternative to antidepressant medications [22] and has demonstrated efficacy, safety, and acceptability in several large-scale multicenter randomized controlled trials (RCTs) [23, 24]. It has also been applied in the treatment of other conditions including schizophrenia, bipolar disorder, and Parkinson’s disease.

The core team of two psychiatrists and two nurses from Ethiopia will undergo intensive and specialized training, including a two-week visit to the CNBS in Munich followed by a regular virtual mentorship and on-site supervision from the team in Munich.

The trained professionals will serve as knowledge multipliers and train other professionals in Ethiopia after acquiring practical experience. The rTMS unit will be established in a dedicated stimulation room at SPHMMC. The trained team is expected to operate the unit for 2.5 h daily, treating approximately five patients per day, with treatment sessions lasting 5–15 min, plus 15 min of preparation. A full treatment series consists of five sessions per week for four weeks. The treatment series may be extended by two additional weeks if a patient shows a positive response, which is defined as a 50% reduction on a validated depression rating scale.

The BEAM project also emphasizes the importance of ensuring the long-term functionality and sustainability of the equipment. To address the uncertainty regarding the acceptance of non-invasive brain stimulation in Ethiopia due to cultural beliefs and stigma, the project will focus on education and dissemination through media and stakeholder engagement. The project aims to transfer knowledge through the trained local stimulation team, and have a representative attend an international conference on brain stimulation.

**Discussion**

The BEAM project aims to introduce rTMS as an innovative treatment modality for severe depression in Ethiopia. The project also addresses important considerations, including the acceptance of non-invasive brain stimulation in the Ethiopian cultural context and the need to address stigma. Ultimately, the BEAM project seeks to expand access to advanced mental health care throughout Ethiopia. It was also discussed that TMS should not be confused with electroconvulsive therapy (ECT).

##### Stratifying recovery within the Ethiopian healthcare system

Mubarak Abera- Ph.D., Professor of Mental Health at Jimma University

**Overview**

Stratifying mental health treatment and recovery involves categorizing patients based on the severity of their conditions, their response to treatment, and the level of care required. This approach ensures that patients receive the most appropriate treatment at the right level of care, optimizing resources and outcomes. This also ensures that resources are used efficiently, that patients receive timely interventions, and that care is personalized to their needs. Recovery stratification also helps to ensure accessibility, continuity of care, comprehensive coverage, and quality care.

The Ethiopian healthcare system is structured into primary, secondary, and tertiary levels.

The primary healthcare level utilizes a workforce of BSc psychiatry nurses, general practitioners, health officers, nurses, and community health workers trained in mental health basics to provide frontline services integrated with general health services. Services include screening, early detection, basic treatment, counseling, prescription of basic psychotropic medications by trained general practitioners, health promotion, and referrals to higher levels. The focus is on mild to moderate conditions with first-line treatments, and response stratification determines whether patients continue treatment at the primary level or are referred to secondary care.

The secondary healthcare level employs psychiatrists, master-level mental health clinicians, clinical psychologists, and social workers to manage moderate to severe mental health conditions. Services include specialist consultations, outpatient services, short-term inpatient care for acute episodes, and coordination with primary care. The focus is on moderate to severe conditions and treatment-resistant cases, with treatment approaches including psychiatric assessments, medication adjustments, short-term psychotherapeutic interventions, and inpatient care for stabilization. Response stratification determines whether patients are stepped down to primary care or require intensified treatment or adjunctive therapies.

The tertiary healthcare level involves highly specialized professionals, providing advanced care for complex and chronic conditions. Services include long-term inpatient care, specialized outpatient clinics, rehabilitation programs, research, and training. The focus is on severe, complex, and treatment-resistant cases, with approaches including advanced psychopharmacological interventions, intensive psychotherapeutic interventions, ECT or TMS, and long-term rehabilitation. Response stratification determines whether patients transition to secondary or primary care for maintenance or require adjustments to advanced therapies.

**Discussion**

In the Ethiopian healthcare system, stratifying recovery entails grouping patients according to the severity of their ailments, how well they respond to treatment, and the degree of care needed to guarantee they get the best care possible while maximizing resource allocation and patient outcomes. Community-Based Mental Health Services provide crisis intervention, peer support, outreach, and reintegration programs, bridging the gap between levels of the health system. When paired with integrated care, monitoring, and feedback, a stepped-care method guarantees smooth patient transitions and comprehensive care. The integration of mental health into primary care, the expansion of training, and the improvement of community-based and telemedicine services are the main answers to the problems of stigma, funding, fragmentation of care, and resource gaps that exist at all levels.

##### Public health strategies for prevention and promotion of mental health

Eshetu Girma- PhD, Research Scientist, Health and Wellbeing at African Population and Health Research Center Nairobi, Kenya (APHRC)

**Overview**

In 1988 Sir Donald Acheson defined public health as the science and art of preventing disease, prolonging life, and promoting health through organized societal efforts. This approach is systemic and holistic, emphasizing community and service-oriented strategies.

Mental health prevention and promotion are critical, with a focus on individual and social capital, along with structural changes. Public health strategies are vital for mental health due to the identified risks and burdens of mental disorders, where behavioral factors are recognized as primary risk factors. It is also essential to understand the core elements of mental health and to promote a shift away from medicalization and hospitalization.

Several challenges hinder mental illness prevention and mental health promotion. These include the climate crisis, environmental degradation, and pollution, as well as poor infrastructure, limited access to services, injustice, discrimination, social exclusion, and the effects of conflict, displacement, and health emergencies. It is difficult to demonstrate the positive impact of preventative actions, and present bias, which prioritizes immediate results over long-term outcomes, also acts as a barrier.

An integrated approach is crucial. This includes having common information systems, professional partnerships with shared responsibilities, and implementing unified policies across physical and mental health. The approach also involves integrating various disciplines such as psychology, neuroscience, sociology, psychiatry, and anthropology, as well as modern and traditional healthcare practices. Additionally, community-based services, digital health initiatives, and addressing social norms play significant roles in achieving better mental health outcomes.

**Discussion**

The presenter emphasized a holistic, systemic approach to mental health, focusing on prevention and promotion through individual, social, and structural strategies. He highlighted the critical role of behavioral factors as primary risks for mental disorders and the need to shift away from medicalization and hospitalization. An integrated, human-centered approach was underscored, involving coordinated policies, professional partnerships, and interdisciplinary collaboration, including psychology, neuroscience, sociology, and traditional practices.

Key components include community-based services, digital health, and addressing social norms. Challenges such as climate change, poor infrastructure, social injustices, and health emergencies were identified as barriers. Key recommendations to improve mental health outcomes include addressing social determinants, engaging communities, leveraging technology, and integrating mental health into broader healthcare systems.

##### The Neurodiversity Center Ethiopia (NDCE)

Tigist Zerihun- MD, MPhil, Assistant prof. of Psychiatry, General and Child and Adolescent psychiatrist SPHMMC, NDCE

**Overview**

The NDCE is a non-profit, board-led organization that focuses on providing comprehensive mental health and neurodivergent community services for infants, children, and adolescents, working in collaboration with governmental and non-governmental institutions.

According to the Federal Ministry of Health Ethiopia (FMOH) 2012 report, mental health issues are prevalent in Ethiopia, with estimates ranging from 12% to 25% of the population. These issues are exacerbated by ongoing civil war, poverty, and infections such as malaria, all of which contribute additionally to mental health challenges. Despite this, most children with mental health conditions do not have access to interventions, highlighting the need for such services.

Currently, there are only three child mental health clinics in the capital city, with no such services in rural areas, and no inpatient facilities available in Ethiopia. The NDCE is actively involved in strengthening infant, child, and adolescent mental health services (ICAMHS) through capacity-building initiatives. These include consultative meetings with various stakeholders, monthly webinars, and teacher training programs, all aimed at raising awareness and knowledge of mental health issues. The center has also developed ICAMH teaching materials, including YouTube videos, moderated by Ethiopian psychiatrists. Basic training in child and adolescent psychiatry, along with assessment tools and advanced clinical training, are provided. Nine child psychiatrists participated in advanced clinical training. Feedback from ICAMH participants is collected and used to improve future courses. Trained professionals from the NDCE now work in various settings such as general hospitals, health centers, orphanages, international schools, NGOs, and tertiary care facilities. Public education is promoted through the media and scholarships.

Outcome measures for the program include tracking referrals, telephonic consultations, pre- and post-tests, and participant feedback. There are also efforts to develop further training and instructional materials, with digital delivery of ICAMH programs being used to disseminate information widely. The program is designed to promote the integration of ICAMS into Ethiopia’s existing healthcare system. The NDCE's efforts are hampered by significant roadblocks, most notably, a lack of funding.

**Discussion**

The NDCE is a board-led, registered non-profit organization that focuses on providing comprehensive ICAMHS services and community support for neurodivergent individuals.

The NDCE is currently trying to develop ICAMH training further and to establish the first inpatient care facility in Ethiopia. The organization emphasizes a multidisciplinary approach and collaboration across various sectors and engages stakeholders like public sector representatives, caregivers, artists, and media professionals. The center also addresses the impact of technology and digital media on children's development and mental health. Despite its efforts, the NDCE faces challenges, such as a lack of professionals and funding. All volunteers who would like to support the center are welcome.

##### The World Health Organization’s Caregiver Skill Training (WHO-CST) program at SPHMMC

Surafel Worku- MD, Assist prof. of psychiatry at SPHMMC and a Ph.D. candidate in Medical Research-International Health at CIH^LMU^

**Overview**

The WHO-CST program aims to address the significant gap in mental health services for children with developmental disabilities (DDs) in Ethiopia, where over 95% of children with DDs reside in LMICs [25]. The core objective of the program is to equip caregivers with essential strategies to support their children’s development. This is achieved through a structured training that includes nine weekly sessions and three home visits. The training focuses on key areas, including enhancing communication and play, promoting adaptive behaviors and learning, reducing challenging behaviors, and prioritizing self-care for the caregivers.

The training has been provided at SPHMMC for more than seven years and more than 200 caregivers graduated from the program. To ensure the long-term sustainability of the program, the training is integrated into the residency curriculum, and 25 facilitators have been trained so far. The program is aligned with the FMOH strategic plan, which emphasizes community-based interventions, empowerment of vulnerable populations, and capacity building.

The feasibility and impact of the program is an ongoing study through both quantitative and qualitative research methods.

Despite its potential, the program faces challenges, including a shortage of space and facilitators, limited training manuals, and the overall burden experienced by caregivers. To overcome these obstacles and expand the program's reach, plans include training more facilitators and caregivers, engaging key stakeholders, and raising community awareness to make the training accessible throughout the country.

**Discussion**

The presentation highlighted the critical need for support for caregivers of children with DDs, particularly in LMICs like Ethiopia, where access to mental health services is severely limited. The long waiting list and parents traveling long distances to attend the training, which demonstrates the critical need for such services The WHO-CST program at SPHMMC is designed to address this gap through a community-based approach. The program has been successfully integrated into the residency curriculum at SPHMMC to ensure its sustainability. The importance of engaging collaborators was also emphasized.

### General recommendations

The following key recommendations are proposed to advance mental healthcare in Ethiopia, based on the presentations and panel discussions across the three thematic areas:

Multi-sectoral integration: Mental health initiatives should be systematically integrated into wider environmental, humanitarian and economic recovery programs (the One Health approach). This recognizes that economic empowerment and ecosystem restoration (e.g., reforestation) can directly strengthen community psychological resilience.

Scale up innovative modalities: Advanced, non-invasive neurostimulation technologies, such as rTMS, should be actively piloted and scaled up in tertiary centers as a sustainable and cost-effective alternative to psychopharmacological supply chains.

Systemic care stratification: A formal stepped-care model must be institutionalized across the primary, secondary and tertiary tires of the Ethiopian healthcare system to optimize scarce mental health resources and streamline referral pathways.

Cultural and community localization: Evidence-based trauma interventions such as NET should utilize local social structures, including traditional elders, religious leaders and indigenous customs such as the coffee ceremony, to facilitate trust and sustainable adaptation.

## Conclusion

The conference highlighted the critical intersection of rehabilitation, innovative strategies, and the One Health framework in addressing mental health challenges, particularly in conflict-affected contexts. After years of restricted travel, it provided an invaluable opportunity to reconnect professionals from across the country, especially from conflict-affected regions, and to create and refresh professional networks. The active participation of key stakeholders demonstrated a collective commitment to advancing mental health care in Ethiopia. This collaboration between CIH^LMU^, i.nez and SPHMMC represents a significant step towards sustainable partnerships that leverage shared expertise and resources. Looking ahead, the insights gained from this conference will play a crucial role in shaping effective mental health recovery strategies, ultimately contributing to the well-being of individuals and communities in Ethiopia and beyond.

## Data Availability

The conference agenda and presentations by conference speakers are available upon request.

## Abbreviations

AAU: Addis Ababa University
AMREF: African Medical and Research Foundation
APHRC: African Population and Health Research Center
BEAM: Brain Stimulation for Ethiopia’s Advanced Mental Health Care
BSc: Bachelor of Science
CBT: Cognitive Behavioral Therapy
CIH: Center for International Health
CIH LMU: Center for International Health Ludwig-Maximilians-University
CMD: Common Mental Disorders
CNBS: Center for Non-invasive Brain Stimulation Munich
DAAD: German Academic Exchange Service (Deutscher Akademischer Austauschdienst)
DDs: Developmental Disabilities
DLPFC: Dorsolateral Prefrontal Cortex
ECT: Electroconvulsive Therapy
EFMOH: Ethiopian Federal Ministry of Health
GBV: Gender-Based Violence
GI: Gastrointestinal
ICAMHS: Infant, Child, and Adolescent Mental Health Services
i.nez: International Network for Cooperation in Mental Health
LMICs: Low and Middle-Income Countries
MA: Master of Arts
MD: Medical Doctor
MPhil: Master of Philosophy
MSc: Master of Science
NET: Narrative Exposure Therapy
NDCE: The Neurodiversity Center Ethiopia
NGO: Non-Governmental Organization
OT: Occupational Therapy
Ph.D.: Doctor of Philosophy
PTSD: Post-Traumatic Stress Disorder
PWLE: People With Lived Experience
RCTs: Randomized Controlled Trials
rTMS: repetitive Transcranial Magnetic Stimulation
SPHMMC: Saint Paul Millennium Medical College
TMS: Transcranial Magnetic Stimulation
UK: United Kingdom
USA: United States of America
WBT: Well-Being Therapy
WHO: World Health Organization
WHO-CST: World Health Organization—Caregiver Skills Training
YLD: Years Lived with Disability
